# Comparison of actual performance in humidification among different high-flow nasal cannula devices: a bench study

**DOI:** 10.3389/fmed.2023.1209977

**Published:** 2023-06-09

**Authors:** Zhong Ni, Yuyan Zhou, Na Tang, He Yu, Zongan Liang

**Affiliations:** Department of Pulmonary and Critical Care Medicine, West China Hospital, Sichuan University, Chengdu, Sichuan, China

**Keywords:** high-flow nasal cannula, flow, humidity, dew point temperature, temperature

## Abstract

**Background:**

The physiological effects of HFNC devices are closely related to temperature and humidity. HFNC devices from different manufacturers may have varied performances. It is unclear whether there are differences in the humidification performance of different HFNC devices and the degree of differences.

**Methods:**

Four integrated HFNC devices (AIRVO 2, Fisher & Paykel Healthcare, Auckland, New Zealand; TNI softFlow 50, TNI Medical AG, Würzburg, Germany; HUMID-BH, RESPIRACARE, Shenyang, China; OH-70C, Micomme, Hunan, China) and a ventilator with an HFNC module (bellavista 1000, Imtmedical, Buchs, Switzerland) were evaluated using their matching circuits. The dew point temperature was set at 31, 34, and 37°C (set-DP). In MR850, it was set to non-invasive mode (34°C/−3°C) and invasive mode (40°C/−3°C), respectively. At each level of set-DP, the flow was set from 20 L/min up to its maximum set limit at a gradient of 5 L/min or 10 L/min. After stabilization, the dew point temperature, temperature, relative humidity, and flow rate of the delivered gas from the cannulas were recorded.

**Results:**

There were significant differences in actual-DP among these devices at any set-DP (*p* < 0.001). The actual-DP of OH-70C and TNI softFlow 50 was lower than set-DP, and the difference between the actual-DP and the set-DP of these two devices increased with the increase of set-DP. AIRVO 2, bellavista 1000 (MR850), and HUMID-BH can provide the nominal humidity at 37°C. The actual-DP increased with the increase of set-flow under each set-DP in AIRVO 2, TNI softFlow 50 and bellavista 1000 (MR850), but decreased when the set-flow was greater than 60 L/min. The actual-T of the delivered gas was higher than actual-DP in all devices and was higher than set-DP in AIRVO 2 and HUMID-BH.

**Conclusion:**

Set-flow, set-DP, and types of devices will affect the actual temperature and humidity of the delivered gas. AIRVO 2, bellavista 1000 (MR850), and HUMID-BH can provide the nominal humidity at 37°C and may be more suitable for tracheotomy patients. The flow rate over 60 L/min should be set with caution.

## 1. Introduction

High-flow nasal cannula (HFNC) oxygen therapy can deliver high flow oxygenated gas ranging from 10 to 80 L/min in adults with the gas adequately heated and humidified. A stable fraction of inspired oxygen (FiO_2_) of 21–100% can be achieved by such a flow. Furthermore, the high flow rate of HFNC can generate positive end-expiratory pressure (PEEP) (1, 2), wash out anatomic dead space in upper airways (3), and decrease the work of breathing (4). Besides, an appropriate gas temperature and humidity can reduce airway contraction, improve mucus-ciliary clearance system function and promote secretion clearance (5), which is another tremendous advantage of HFNC. Dry or poorly humidified medical gases may reduce epithelial cell function, increase inflammation, and cause dry nose, pharynx, and nasal pain, which can provoke respiratory tract spasms and increase airway resistance (6–9). The use of HFNC can prevent nasal epistaxis (5, 6). HFNC has been widely concerned as a non-invasive respiratory support alternative in critically ill patients as many studies have proved that HFNC has some advantages over conventional oxygen therapy (COT) and non-invasive ventilation (NIV) (10–13). A recent study confirmed that HFNC could improve the dyspnea score and mucus production in acute exacerbation of chronic obstructive pulmonary disease patients (14). Furthermore, the heat and humidification delivered by HFNC help to maintain hydration and mobilize secretions, which positively affect the mucus hypersecretion of patients with COVID-19 (15).

There are many kinds of HFNC devices on the market, including high-flow air oxygen mixer systems, air entrainment systems, and integrated HFNC devices (16). In addition, some manufacturers have added HFNC modules for ventilators. To reduce condensation, most HFNC devices were equipped with servo-controlled heating wires to the circuit. However, condensation cannot be avoided entirely. The international standard for HFNC devices (ISO/DIS 80601-2-90) was recently published and updated by the International Organization for Standardization (17). This document addresses the basic safety and essential performance requirements of respiratory high-flow therapy equipment. The absolute humidity must not be lower than 16 mg/L (or equivalent to 90% relative humidity at 22°C) for use via nasal cannulas and shall not be lower than 33 mg/L for use in patients with upper respiratory tract bypass. The output gas of the humidifier should not exceed 43°C to avoid being burned. The differences in the length, position, and shape of the heating wire may end up in different outputs under identical settings, which might affect the clinical assessment. A previous study related to heat and moisture exchangers showed that manufacturer specifications and bedside measurements of absolute humidity differed considerably for the Hygroster, which did not achieve efficient humidification in certain instances (18). So, an independent assessment is required. A few studies in this field explored how parameter settings affect humidification performance, condensation, and patient tolerance in models and human volunteers (19–23).

According to previous studies, flow plays a vital role in the humidification performance of HFNC (24–27). This study aims to explore the characteristics and limits of humidification performance among different devices in real ward environment by measuring actual-T and humidity at the nasal cannulas under various flow settings. This study may help clinicians with a profound understanding of the differences between actual output and set parameters, as well as the differences between different HFNC devices. Moreover, the results may provide a basis for the selection and parameter setting of different devices.

## 2. Materials and methods

**Figure 1:**
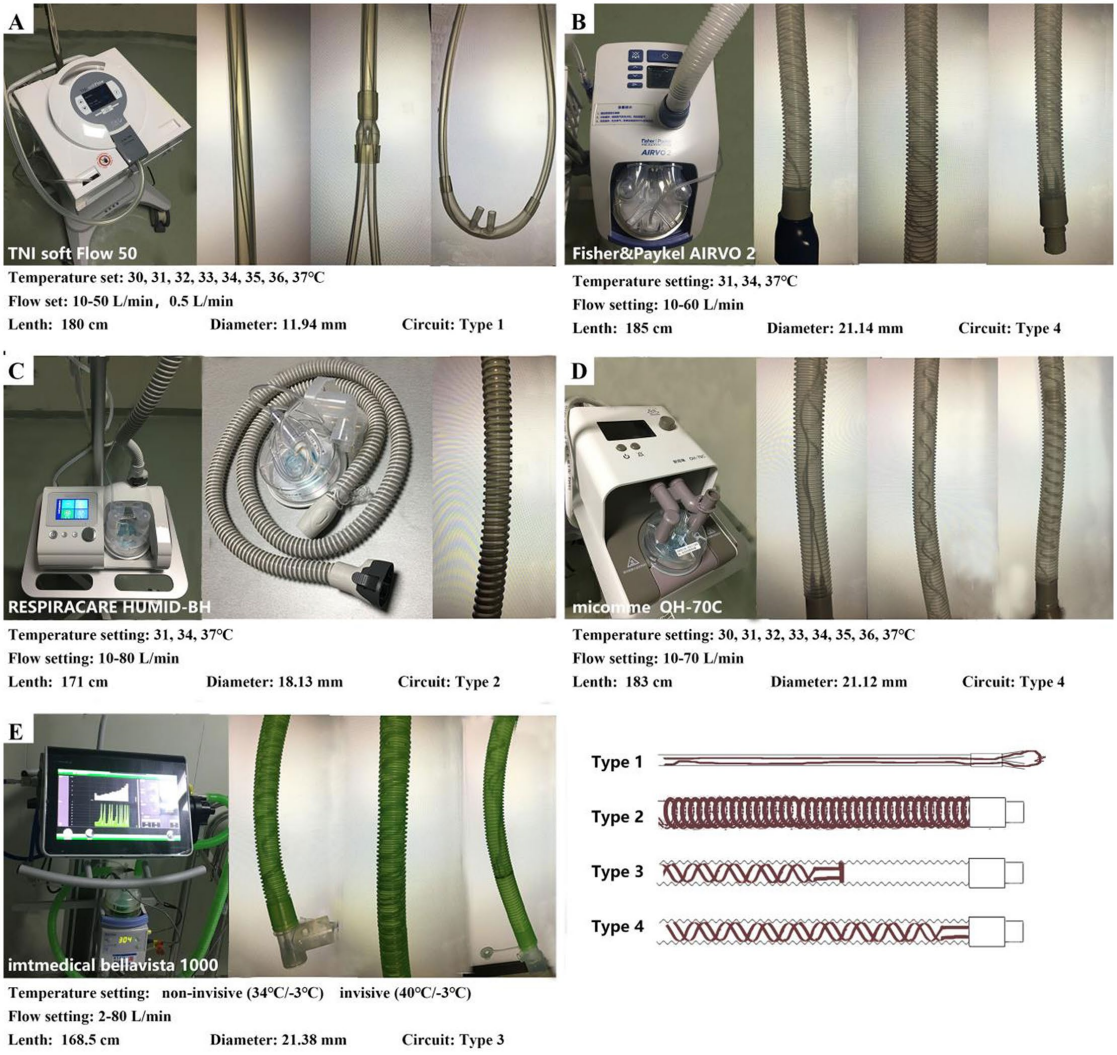
Settings of the HFNC devices and characteristics of the circuits. **(A)** TNI softFlow 50: The diameter of the circuit is smaller than other devices, and the heating wire is straight and runs through the whole circuit and the nasal cannulas (Type 1). **(B,D)** AIRVO 2 and OH-70C: the heating wire is arranged in a double helix structure in the whole circuit (Type 4). **(C)** HUMID-BH: The heating wire is wrapped in a spiral shape outside the circuit (Type 2). **(E)** Bellavista 1000 (MR850): the heating wire is arranged in a double helix structure in the circuit and located approximately 3/4 of the circuit near the humidifier (Type 3). Temperature sensors are installed both at the chamber outlet and the end of the heating circuit to provide feedback on the temperature and humidity output of MR850 in real time.

We tested four integrated HFNC devices (AIRVO 2, Fisher & Paykel Healthcare, Auckland, New Zealand; TNI softFlow 50, TNI Medical AG, Würzburg, Germany; HUMID-BH, RESPIRACARE, Shenyang, China; OH-70C, Micomme, Hunan, China) and a ventilator with an HFNC module (bellavista 1000, Imtmedical, Buchs, Switzerland) using their matching heating circuits, humidification chambers, nasal cannulas, and other accessories. The devices were provided by the West China Hospital’s Medical Intensive Care Unit (MICU) and have been used stably in the clinic without fault. Figure 1 shows the detailed information and appearance of the devices and heating circuits.

A multi-function measuring instrument ALMEMO ®2,390-8 with a fixed digital hygrometer FNAD46-3 was used to measure the temperature, dew point, and relative humidity of actually delivered gas.

### 2.1. Preparations

**Figure 2:**
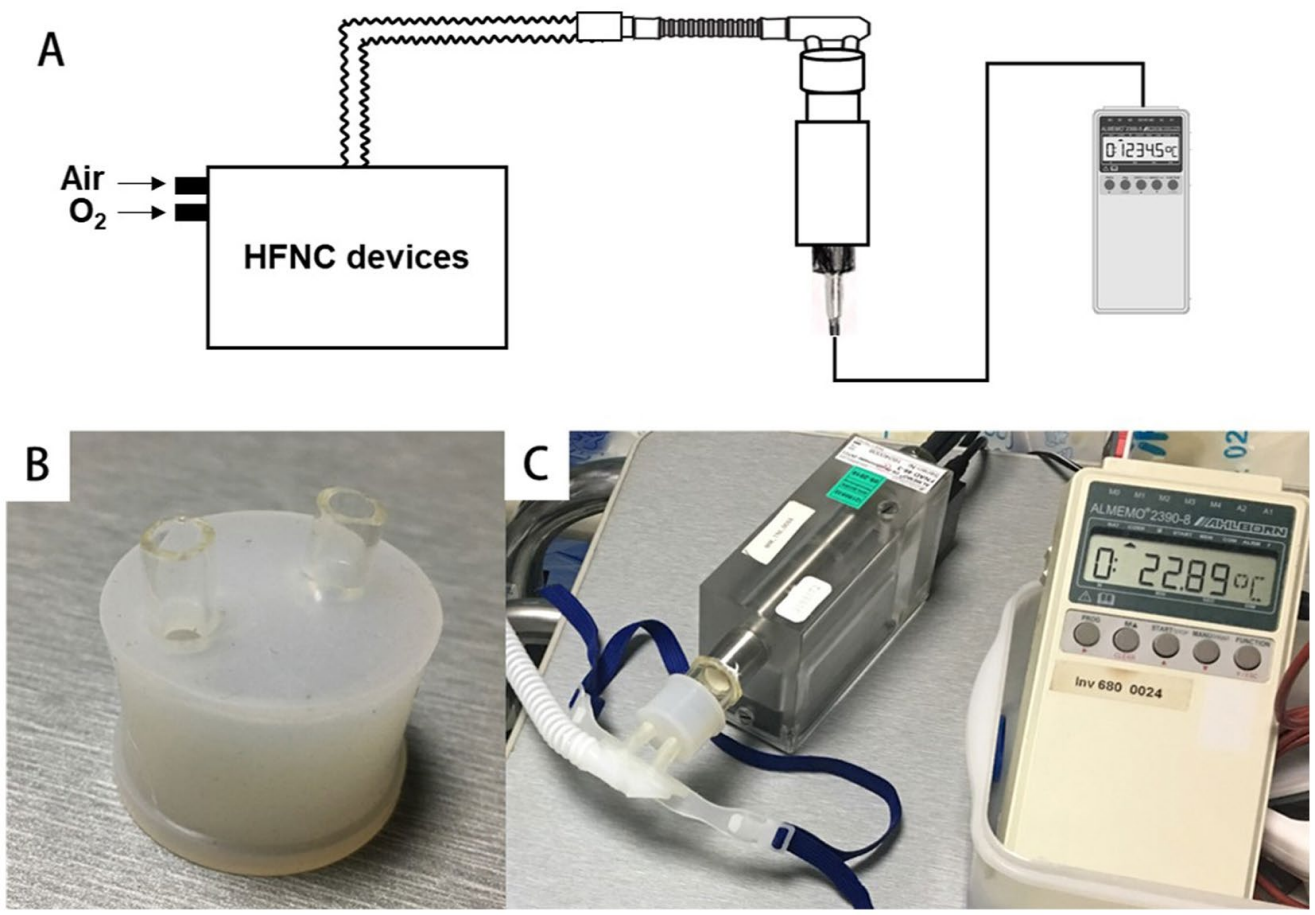
Connect the cannulas to the digital hygrometer via a special adapter. **(A,C)** The schematic diagram and real scene of the connection between the equipment and the hygrometer. **(B)** The special adapter.

As shown in Figure 2, the cannulas were connected to the measuring instrument, so the target parameters at the nasal plug are measured directly using the same adapter as the study (28).

The measurement was carried out in an independent ward (4 m long, 3 m wide, and 2.5 m height, with no strong air convection) in the MICU of West China Hospital. The ambient temperature was controlled, and all equipment that might interfere with the test was removed from the ward. All HFNC devices and measuring instruments were fully preheated and calibrated before measurement. During the testing process, the ambient temperature was 22.1 ± 0.7C, the relative humidity was 74 ± 7%, and the atmospheric pressure was 716.9 ± 5.1 mmHg.

### 2.2. Protocol

The dew point or dew point temperature of a gas is the temperature at which the water vapor contained in the gas is transformed into a liquid state. The water vapor contained in the gas exists as a liquid below the dew point temperature, while a gaseous component is above the dew point temperature. For example, the dew point temperature of 20°C can be equated with absolute humidity 16 mg/L (minimum requirements of the ISO standard) at standard atmospheric pressure. So, the dew point or dew point temperature is an indicator of absolute humidity, and three of the tested devices (AIRVO 2, TNI softFlow 50, and HUMID-BH) take the dew point temperature as the temperature setting target. Therefore, we choose the dew point temperature to reflex the humidification ability of the devices.

The dew point temperature was set at 31, 34, and 37°C, named set-DP. In MR850, it was set to non-invasive mode (34°C /−3°C) and invasive mode (40°C/−3°C), respectively. At each level of set-DP, the flow was set at 20, 25, 30, 35, 40, 45, 50, 60, 70, and 80 L/min, depending on their maximum flow, which was named set-flow. The FiO_2_ was fixed at 21%.

The dew point temperature, temperature, relative humidity, and flow rate of the delivered gas from the cannulas were named actual-DP, actual-T, actual-RH, and actual-flow. For each setting, after stabilization for 15 min, actual-DP and actual-flow were recorded three times at an interval of 10 s, respectively. The test was repeated with set-flow increasing and decreasing orders to reduce the influence of heat accumulation. And then all the testing processes were repeated twice at different date to reduce disturbance from possible environmental factors and avoid contingency.

### 2.3. Statistics

Finished testing for normality, nonnormally distributed variables are expressed as medians (interquartile range), and normally distributed variables are expressed as the mean ± SD. Kruskal-Wallis H test was used to compare the effect of different set-flow on actual-T and actual-DP in a single device. Kruskal-Wallis H test was also used to compare actual parameters in different systems with the same settings. Wilcoxon signed rank test was used to compare differences between settings and actual parameters. The statistical tests were two-sided, and *p* < 0.05 was considered statistically significant. All statistical analyses were performed using IBM SPSS statistical software.

## 3. Results

There was no significant difference in actual-T and actual-DP between the flow-increasing and the flow-decreasing setting groups (*p* = 0.117 and 0.325). The test order and the running time of the devices do not affect actual-T and humidification efficiency. The following analysis no longer considers the test order.

### 3.1. Difference between actual-DP and set-DP in a single device and different devices

**Figure 3:**
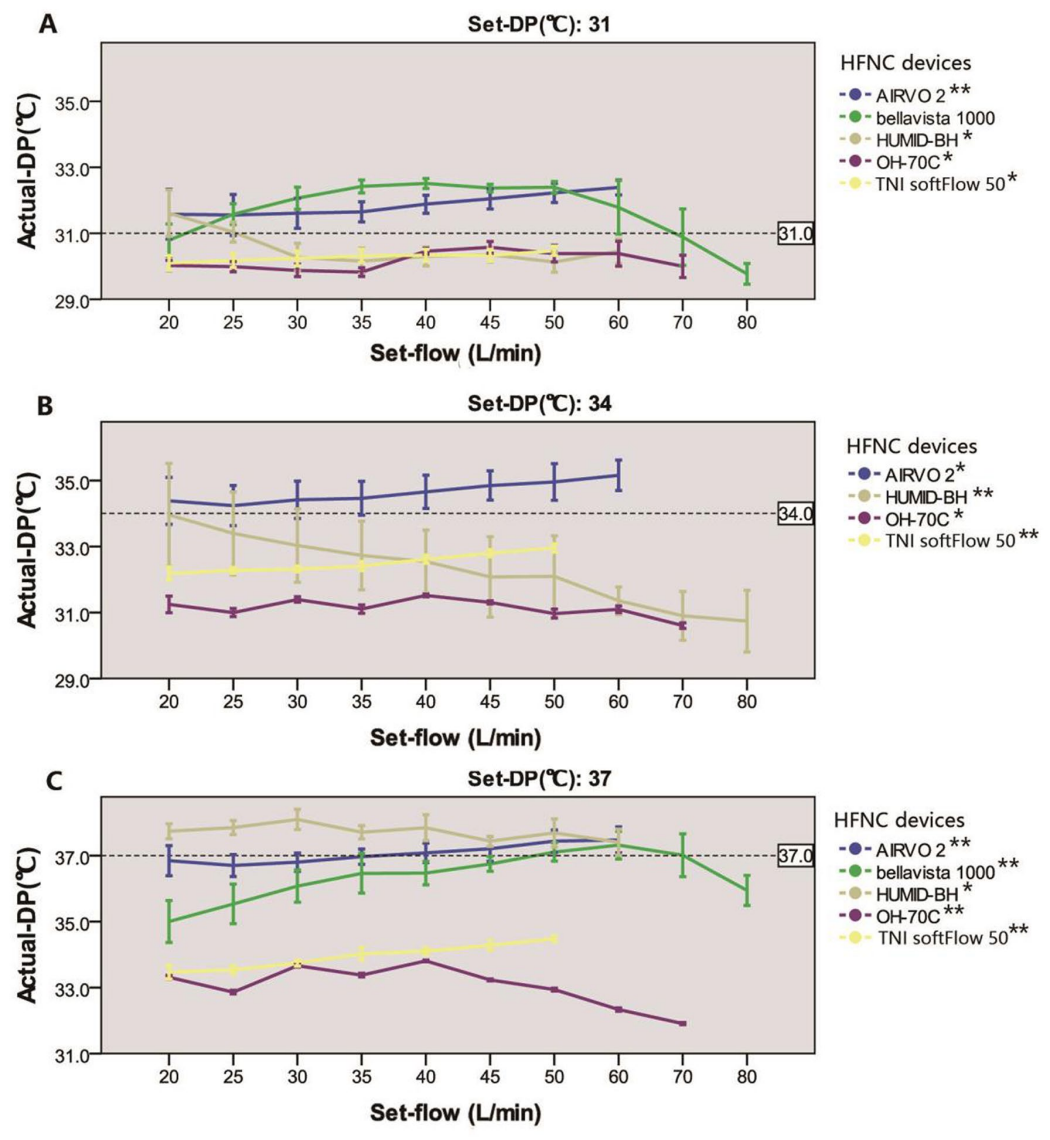
Relationship between actual-DP and set-flow levels in five devices at set-DP 31°C **(A)**, 34°C **(B)**, and 37°C **(C)**. *There was a significant correlation between actual-DP and the set-flow at this set-DP (*p* < 0.001). The correlation coefficient was between 0.3 and 0.5; **The correlation coefficient was larger than 0.5. The error line represents the standard deviation.

Actual-DP of the five devices in the real ward environment was summarized in Table 1. There were significant differences in actual-DP among these devices at any set-DP (*p* < 0.001). Besides, there were significant differences between actual-DP and the set-DP of all the tested device (*p* < 0.001) except for AIRVO 2 when the dew point was set at 37°C (Table 1). Table 1 and Figure 3 showed that the deviation of the actual-DP of OH-70C and TNI softFlow 50 increased with increasing set-DP. Actual-DP of these two devices was lower than the corresponding set-DP and was considerably lower when the dew point was set at 37°C. AIRVO 2, bellavista 1000 (MR850), and HUMID-BH can provide enough humidity at set-DP 37°C.

**Table 1:** Characteristics of actual-DP in five devices.

Devices	Actual-DP (°C)		
	Set-DP: 31°C	Set-DP: 34°C	Set-DP: 37°C
AIRVO 2	31.94 (31.43, 32.25)	34.71 (34.08, 35.14)	37.00 (36.88, 37.31)^*^
TNI softFlow 50	30.28 ± 0.22	32.48 (32.24, 32.77)	33.98 (33.63, 34.27)
HUMID-BH	30.51 (30.08, 30.90)	31.85 (31.41, 33.75)	37.72 ± 0.36
OH-70C	30.17 ± 0.34	31.14 (30.96, 31.35)	33.22 (32.81, 33.43)
Bellavista 1000 (MR850)	32.10 (30.98, 32.43)	N/A	36.47 (35.73, 36.95)

The data are presented as the median (IQR) or mean ± SD. *There was no significant difference between the actual-DP and the corresponding set-DP (*p* = 0.134).

### 3.2. Influence of set-flow on actual-DP in a single device

There is no difference between actual-DP and the set-DP in AIRVO 2 when set-flow 35–45 L/min at set-DP 37°C. In other devices, there were significant differences in actual-DP at different set-flow rates under the same set-DP (*p* < 0.001). Except for bellavista 1000 (MR850) at set-DP 31°C, there was a significant correlation between actual-DP and set-flow in all devices at the same set-DP (*p* < 0.001). Under each set-DP, actual-DP in AIRVO 2 and TNI softFlow 50 showed an increasing trend with increased set-flow. Actual-DP in bellavista 1000 (MR850) increased with the rise of set-flow when set-flow was lower than 60 L/min and decreased with the increase of set-flow when it exceeded 60 L/min.

### 3.3. Actual-T and actual-RH in a single device and different systems

**Figure 4:**
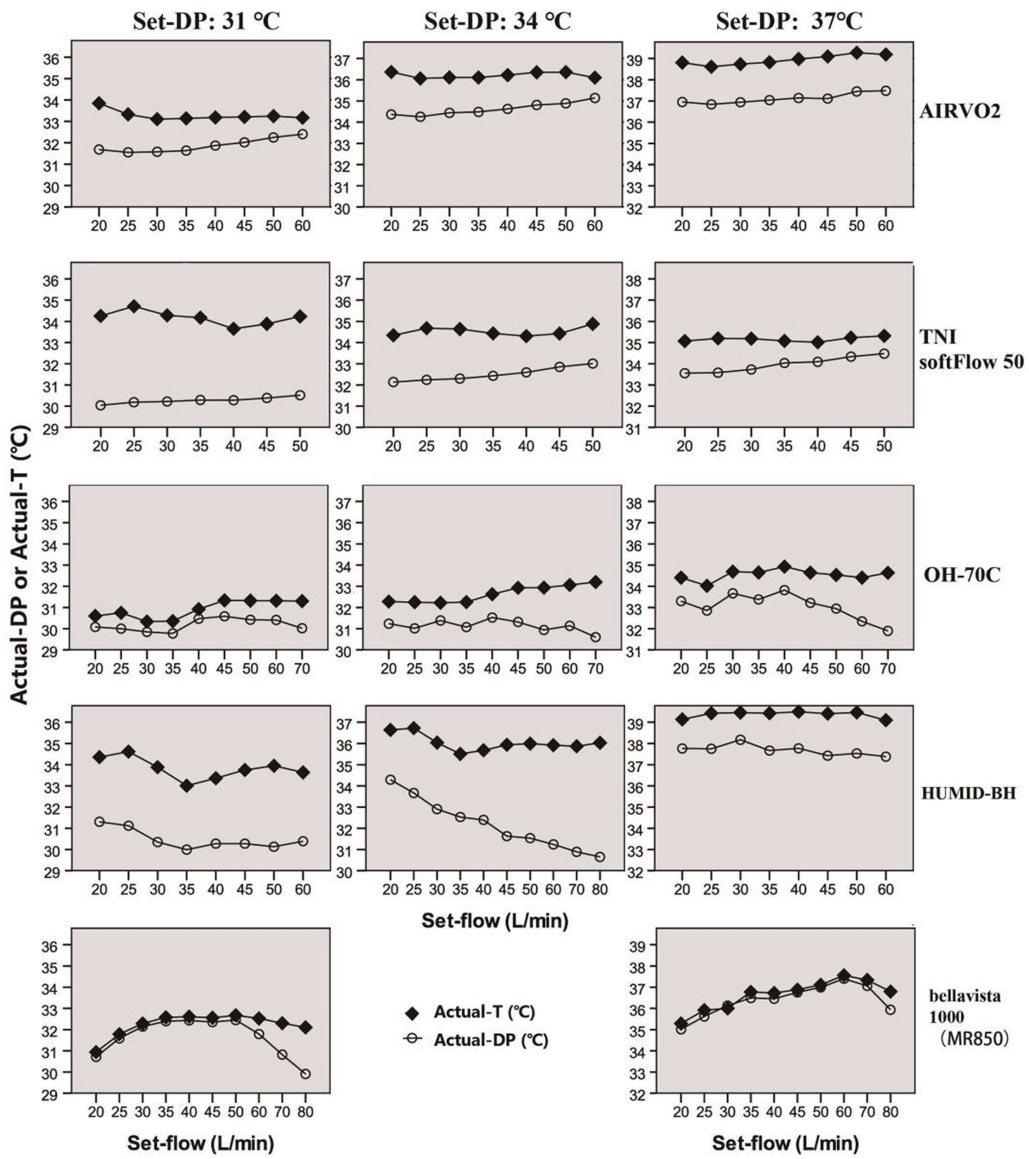
Actual-T and actual-DP of five devices at different set-flow levels under different set-DPs.

The actual-T and actual-RH of the five devices in the real ward environment were summarized in Tables 2, 3 and Figure 4. The actual-T of bellavista1000 (MR850) was close to the set-DP when set-flow below 60 L/min. When set-flow was higher than 60 L/min, the difference between the actual-T and set-DP increased with increasing set-flow. Bellavista1000 (MR850) can provide the target actual-T and the highest actual-RH.

**Table 2:** Characteristics of actual-T in five devices.

Devices	Actual-T (°C)		
	Set-DP: 31°C	Set-DP: 34°C	Set-DP: 37°C
AIRVO 2	33.23 (33.08, 33.37)	36.20 (36.01, 36.61)	38.93 (38.75, 39.13)
TNI softFlow 50	34.21 (33.68, 34.77)	34.53 (34.32, 34.91)	35.16 (35.03, 35.27)
HUMID-BH	33.81 (33.33, 34.13)	35.98 (35.72, 36.14)	39.43 (38.95, 39.77)
OH-70C	30.92 (30.51, 31.32)	32.61 (32.25, 32.96)	34.57 (34.33, 34.80)
Bellavista 1000 (MR850)	32.36 (32.06, 32.61)		36.90 (36.27, 37.25)

The data are presented as the median (IQR) or mean ± SD.

**Table 3:** Characteristics of actual-RH in five devices.

Devices	Actual-RH		
	Set-DP:31°C	Set-DP:34°C	Set-DP:37°C
AIRVO 2	90.4% ± 3.2%	89.6% ± 2.3%	88.7% ± 1.4%
TNI softFlow 50	75.3% (74.0, 78.3%)	86.7% ± 2.3%	92.6% (90.4, 94.3%)
HUMID-BH	79.2% (77.5, 81.5%)	75.0% (72.9, 85.4%)	90.3% (85.9, 94.5%)
OH-70C	95.2% (93.4, 96.2%)	90.5% (87.0, 93.1%)	91.4% (88.3, 93.0%)
Bellavista 1000 (MR850)	98.4% (97.5, 98.9%)		98.2% (97.9, 98.7%)

The data are presented as the median (IQR) or mean ± SD.

By contrast, the actual-T of the integrated HFNC devices was generally higher than set-DP, while the actual-DP was lower than set-DP. The actual-T of HUMID-BH could reach 39.43(38.95, 39.77) °C when the dew point was set at 37°C.

## 4. Discussion

In this study, the actual output of five devices under different settings was quantitatively analyzed. The results showed that actual-T and dew point varied depending on the set-DP, set-flow, and the devices. In addition, three points are worth noting: (1) AIRVO 2, bellavista 1000 (MR850), and HUMID-BH can provide the target humidity, especially when the dew point temperature was set at 37°C, (2) However, OH-70C and TNI softFlow 50 cannot provide the actual-DP exceeding 34°C, and (3) The actual-T of OH-70C and TNI softFlow 50 was significantly lower than the set-DP.

AIRVO 2, TNI softFlow 50, and bellavista 1000 (MR850) all present that the actual-DP increases with the increase of set-flow. The integrated HFNC devices optimize the cooperative control of temperature, humidity, and flow output, which can automatically adjust the power of the heating plate according to set-flow, so as to ensure the appropriate humidification effect and avoid the occurrence of insufficient or excessive humidification. MR850 monitors the temperature at the outlet of the humidification chamber and the end of the heating circuit in real time, and the feedback circuit controls the power of the heating plate and heating wire to maintain a stable temperature and humidity output. Several studies on AIRVO 2 and MR850 have obtained the same results (23, 25, 29). Plotniko et al. (27) reported that the best performance for all flows in terms of AH was found with the Fisher & Paykel MR850 HH, regardless of the circuit used. According to their analysis, in addition to the corresponding increase in heating power, when the set-flow increases, the heat exchange time between the gas and the ambient is shorter, so the condensation loss is less and the humidity is higher.

The actual-DP of bellavista 1000 (MR850) decreased with the increase of set-flow when set-flow was greater than 60 L/min. Actual-DP of HUMID-BH decreased obviously with the increase of flow rate when the dew point was set at 34°C (the flow rate of HUMID-BH can only be set to 60–80 L/min when DP is set at 34°C due to the limit of the device). The gas passes over a heated water chamber faster at a higher flow rate (>60 L/min) and the insufficient power of the heating humidifier may lead to a decline in humidification efficiency. Chikata et al. (23) monitored the electrical output of the heating plate of MR850 using View850 (Fisher & Paykel). It was proved that the output power of the heating plate was less than 100% when the flow was set at 20 and 40 L/min. When the flow was set at 60 L/min, the electric output of the heating plate was maintained at 100%, and the temperature at the outlet of the humidifier and at the end of the circuit was lower than set. A study on the humidification efficiency of HFNC at a higher flow rate (60–100 L/min) confirmed the same results that traditional heating humidifiers could not provide sufficient humidification at 60–100 L/min (24). Plotnikow et al. (27) got the same results. On the other hand, previous studies showed that when the patient’s inspiratory flow exceeded the HFNC flow, the absolute humidity of the inhaled gas was affected by tidal volume (25). Meanwhile, studies have shown that a higher flow rate (60 L/min) can improve patients’ comfort with severe hypoxemia (30). Another study on healthy volunteers confirmed they preferred flow delivered at 30 or 40 mg H_2_O/L (19). Therefore, the heating and humidification of higher flow rate gas still deserves attention and needs to be improved.

The actual-T of independent HFNC devices was higher than the corresponding actual-DP and was higher than the set-DP in some devices. The actual-T of bellavista 1000 (MR850) is similar to the corresponding actual-DP. Accordingly, the condensation of bellavista 1000 (MR850) was remarkable. To minimize condensation, manufacturers usually set the heating wire temperature 2–3°C higher than the set-DP. When set to invasive mode (40°C/−3°C), the MR850 humidifier will heat the gas to 37°C and relative humidity 100% at the outlet of the humidification chamber. Then the gas will be heated to 40°C in the heating circuit and maintained at 40°C during transportation (relative humidity<100%). The temperature sensors and servo-control system achieve the stable temperature and humidity output of MR850 (Figure 1E). When it reaches the nasal cannulas section (without heating guide wire), the gas will be cooled to 37°C and RH100% again at the nasal cannulas. Independent HFNC device also adopts such a design. However, the temperature of the heating wire might be too high to cool down to the target temperature due to the lack of servo-control. At this time, the humidity of the delivered gas is unsaturated, and condensation is not easy to occur, which is consistent with the observed results in the study. But there is a problem that the comfort of patients may be reduced. Chikata et al. explored the influence of room temperature and the covering length of the heating wire on condensation. They found that reducing the internal and external temperature difference of the circuit and increasing the length of the heating guide wire can reduce condensation effectively (29, 31). According to the characteristics of the circuit, the actual-T of TNI softFlow 50 (small diameter and straight heating wire in the whole circuit and the nasal cannulas, Figure 1A) should be the highest among the five devices tested theoretically. But the results show that actual-T was 34.21 (33.68–34.77), 34.53(34.32–34.91) and 35.16(35.03–35.27) °C, respectively, when DP was set at 31,34,37°C. In OH-70C (double helix heating wire in the whole circuit, Figure 1D), the actual-T was lower than set-DP. In HUMID-BH (heating wire wrapped outside the circuit, Figure 1C), the actual-T was the actual highest, which might be related to the slow heat dissipation. The circuit characteristics of the five devices tested in this study are not significantly related to the heating and humidification efficiency. The working power and the algorithm of the devices are the main factors that affect this result, in addition to the circuit structure. So, the advantages and disadvantages of different structures cannot be compared, and further research is needed.

The high temperature of gas delivered by HFNC devices is one of the reasons why patients do not tolerate HFNC treatment. A previous study found that patients who failed HFNC had a higher incidence of discomfort 1 h after initiation (32), suggesting that the comfort may be related to clinical outcomes. However, the optimum humidity and temperature of HFNC are still controversial. The default setting of 37°C can achieve the best humidification efficiency. But there is no evidence to prove that 37°C is the best for HFNC because of the retained humidification function of the upper respiratory tract. The results of this study show that actual-T in most devices is relatively higher than set-DP, and the setting of 37°C might not be conducive to patient comfort. At present, no studies have provided evidence for the effect of actual-T of HFNC on patients’ comfort. Only a short time (20 min) cross-test was conducted concerning the set-DP, and the results showed that the lower set temperature was related to higher comfort (30). The study also claimed that the results were highly heterogeneous.

This study provides data for evaluating the actual performance of HFNC devices in the real ward environment. The differences between set values and actual values exist objectively, and more bench and clinical studies are needed to determine and quantify the consequences of these differences. Increasing the number of devices to be tested, improving the airway model and spontaneous breathing simulation in bench studies, or conducting clinical tests on healthy volunteers or representative patients are all essential directions in future research.

This study has several limitations. Firstly, the result can only represent the actual output under the specific environmental conditions in this study, and it may change in different environments. Secondly, we only tested one device from each manufacturer, and there are inevitably individual differences. Thirdly, although all the tests we conducted in a single room which did not have strong air convection and were controlled by an air conditioning system, it was impossible to maintain absolutely constant ambient temperature and humidity, which may also affect the results of the tests.

## 5. Conclusion

Set-flow, set-DP, and types of devices will affect the actual temperature and humidity of the delivered gas. And there is a deviation from the nominal output with uncertain clinical consequences. AIRVO 2, bellavista 1000 (MR850), and HUMID-BH can provide the nominal humidity at 37°C and may be more suitable for tracheotomy patients. The flow rate over 60 L/min should be set with caution, in which situation both the built-in heating humidifier in HFNC devices and the traditional independent heating humidifier failed to provide sufficient humidity. The actual-T of the delivered gas is higher than actual-DP or set-DP, which may cause the patient discomfort.

## Data availability statement

The original contributions presented in the study are included in the article/supplementary material, further inquiries can be directed to the corresponding authors.

## Author contributions

ZN and YZ designed the study, drafted the manuscript, and conducted the literature search and data analysis, contributing equally to the study. NT assisted with experiments and manuscript preparation. ZL and HY revised the manuscript critically for important intellectual content, and gave the final version for publication. All authors contributed to the article and approved the submitted version.

## Conflict of interest

The authors declare that the research was conducted in the absence of any commercial or financial relationships that could be construed as a potential conflict of interest.

## Publisher’s note

All claims expressed in this article are solely those of the authors and do not necessarily represent those of their affiliated organizations, or those of the publisher, the editors and the reviewers. Any product that may be evaluated in this article, or claim that may be made by its manufacturer, is not guaranteed or endorsed by the publisher.
